# Developing a Predictive Model for Significant Prostate Cancer Detection in Prostatic Biopsies from Seven Clinical Variables: Is Machine Learning Superior to Logistic Regression?

**DOI:** 10.3390/cancers17071101

**Published:** 2025-03-25

**Authors:** Juan Morote, Berta Miró, Patricia Hernando, Nahuel Paesano, Natàlia Picola, Jesús Muñoz-Rodriguez, Xavier Ruiz-Plazas, Marta V. Muñoz-Rivero, Ana Celma, Gemma García-de Manuel, Pol Servian, José M. Abascal, Enrique Trilla, Olga Méndez

**Affiliations:** 1 Department of Urology, Vall Hebron University Hospital, 08035 Barcelona, Spain; ana.celma@vallhebron.cat (A.C.); enrique.trilla@vallhebron.cat (E.T.); 2 Department of Surgery, Universitat Autònoma de Barcelona, 08193 Bellaterra, Spain; npaesa@gmail.com; 3 Research Group in Urology, Vall Hebron Research Institute, 08035 Barcelona, Spain; 4 Statistics and Bioinformatics Unit, Vall d’Hebron Research Institute, 08035 Barcelona, Spain; berta.miro@vhir.org; 5 Department of Artificial Intelligence and Big Data, GMV Innovative Solutions Inc., 28760 Madrid, Spain; patricia.hernando.sanchez@gmv.com; 6 Clínica Creu Blanca, 08018 Barcelona, Spain; 7 Department of Urology, Hospital Universitari de Bellvitge, 08907 Hospitalet de Llobregat, Spain; npicola.bellvitge@gencat.cat; 8 Department of Urology, Hospital Universitari Parc Tauli, 08208 Sabadell, Spain; jmunoz@tauli.cat; 9 Department of Urology, Hospital Universitari Joan XXIII, 43005 Tarragona, Spain; xruiz.tarragona.ics@gencat.cat; 10 Department of Urology, Hospital Universitari Arnau de Vilanova, 25198 Lleida, Spain; mvmunoz.lleida.ics@gencat.cat; 11 Department of Urology, Hospital Universitari Josep Trueta, 17007 Girona, Spain; gemmagarcia.girona.ics@gencat.cat; 12 Department of Urology, Hospital Universitari Germans Trias i Pujol, 08916 Badalona, Spain; pservian.germanstrias@gencat.cat; 13 Department of Urology, Parc de Salut Mar, 08003 Barcelona, Spain; jabascal@psmar.cat; 14 Department of Medicine and Health Sciences, Universitat Pompeu Fabra, 08002 Barcelona, Spain

**Keywords:** predictive models, prostate cancer detection, machine learning, logistic regression

## Abstract

**Objective**: This study compares machine learning (ML) and logistic regression (LR) algorithms in developing a predictive model for sPCa using the seven predictive variables from the Barcelona (BCN-MRI) predictive model. **Method**: A cohort of 5005 men suspected of having PCa who underwent MRI and targeted and/or systematic biopsies was used for training, testing, and validation. A feedforward neural network (FNN)-based SimpleNet model (GMV) and a logistic regression-based model (BCN) were developed. The models were evaluated for discrimination ability, precision–recall, net benefit, and clinical utility. Both models demonstrated strong predictive performance. **Results**: The GMV model achieved an area under the curve of 0.88 in training and 0.85 in test cohorts (95% CI: 0.83–0.90), while the BCN model reached 0.85 and 0.84 (95% CI: 0.82–0.87), respectively (*p* > 0.05). The GMV model exhibited higher recall, making it more suitable for clinical scenarios prioritizing sensitivity, whereas the BCN model demonstrated higher precision and specificity, optimizing the reduction of unnecessary biopsies. Both models provided similar clinical benefit over biopsying all men, reducing unnecessary procedures by 27.5–29% and 27–27.5% of prostate biopsies at 95% sensitivity, respectively (*p* > 0.05). **Conclusions**: Our findings suggest that both ML and LR models offer high accuracy in sPCa detection, with ML exhibiting superior recall and LR optimizing specificity. These results highlight the need for model selection based on clinical priorities.

## Simple Summary

Prostate cancer (PCa) detection remains a critical area of research, with an ongoing need for predictive tools that accurately identify significant PCa (sPCa) while decreasing unnecessary prostate biopsies and the overdetection of insignificant tumors. Risk calculators based on predictive models are among the most valuable tools, as they can individualize the likelihood of sPCa with high accuracy at no cost. Machine learning algorithms are the modern preferred methods for developing predictive models, especially when managing big data. However, it remains unclear whether machine learning is superior to traditional logistic regression. In this study, we demonstrate that both algorithms proved similarly effective on a limited dataset.

## 1. Introduction

Prostate cancer (PCa) screening is currently recommended by the European Union based on compelling evidence supporting its benefits [1]. The European Randomized Screening for Prostate Cancer (ERSPC) has been instrumental in shaping these guidelines. The screened group demonstrated a 20% reduction in PCa-specific mortality compared to the control group after a median follow-up of eight years [2]. Long-term benefits have also been observed over 22 years in the Göteborg randomized screening program, further highlighting the sustained impact of PCa screening on mortality reduction [3].

Recent advancements in PCa screening focus on identifying significant PCa (sPCa) while minimizing unnecessary prostate biopsies and reducing the detection of insignificant PCa (iPCa). This approach integrates elevated serum prostate-specific antigen (PSA) levels with advanced imaging techniques such as magnetic resonance imaging (MRI). MRI facilitates risk stratification of sPCa in suspicious lesions using the Prostate Imaging-Reporting and Data System (PI-RADS). The integration of MRI with transrectal ultrasound (TRUS) via fusion imaging technology enhances the accuracy of targeted biopsies with PI-RADS v2.1 further improving diagnostic efficacy [4,5]. However, given MRI’s limited positive predictive value, particularly in cases with a PI-RADS score of 3, additional tools to refine biopsy decisions are needed [6,7]. PSA density, modern biomarkers, and novel predictive models offer promising strategies to improve biopsy candidate selection and optimize diagnostic strategies [8,9,10,11,12].

The Barcelona-MRI (BCN-MRI) predictive model was developed to enhance sPCa risk assessment by improving its detection in prostate biopsies. This model was developed using logistic regression (LR) and seven independent predictive clinical variables after MRI: age (years), type of biopsy (initial vs. repeated), PCa family history (no vs. yes), serum PSA level (ng/mL), digital rectal examination (DRE: normal vs suspicious), MRI-based prostate volume (mL), and PI-RADS v2.0 score (1–5) [12]. Although LR remains widely used and accessible, it has become increasingly outdated due to evolving clinical practices, requiring external validations to maintain its relevance [13]. In contrast, machine learning (ML) models offer greater adaptability by continuously integrating new cases and outcomes, as well as big data management. These models can accommodate diverse data inputs, including genomic and radiomic data, enhancing predictive accuracy and clinical applicability [14,15]. Additionally, ML algorithms facilitate federated networks, reducing the need for repeated model validation across different clinical sites. This capability may improve the generalizability and robustness of sPCa predictions across diverse populations [16]. The feedforward neural network (FNN)-based SimpleNet model builds upon MRI-based predictive modeling for sPCa, proposing that ML-driven predictions may surpass LR in clinical effectiveness [17]. FNNs, known for their effectiveness in modeling complex nonlinear relationships, offer a robust framework for integrating and analyzing structured clinical and biomarker data to enhance predictive modeling in sPCa [18].

The effectiveness of predictive models developed with ML and LR methods, in different areas of health, has been recently compared with controverted results [19,20,21,22,23,24]. We hypothesize that an ML-based model for sPCa detection will demonstrate superior predictive performance compared to an LR-based model, particularly in terms of sensitivity and overall clinical utility. Our objective is to compare the clinical effectiveness of a novel SimpleNet FFN-based predictive model, developed by GMV Innovative Solutions Inc., Madrid, Spain, with a traditional LR-based model (BCN) for sPCa detection.

## 2. Materials and Methods

### 2.1. Study Design and Participants

This was a retrospective study conducted among 5005 men suspected of having prostate recruited at ten participant centers of the sPCa opportunistic screening program in Catalonia, Spain. The inclusion criteria were to have undergone a pre-biopsy multiparametric MRI (mpMRI) and targeted and/or systematic prostate biopsy between 1 January 2016, and 31 December 2022. Exclusion criteria were to have had a previous PCa diagnosis or atypia, and a lack of reporting of the seven clinical predictive variables included in the BCN-MRI predictive model. This study was approved by the ethics committee of the coordinator center (PRAG-02/2021, approved on 12 February 2021).

### 2.2. Diagnostic Approach for Significant Prostate Cancer

A serum PSA level higher than 3.0 ng/mL and/or a suspicious DRE were detected at the primary healthcare center, leading to suspicion of PCa [25]. These men were referred to their reference center where mpMRI exams were conducted, using 1.5 or 3 Tesla scans with a pelvic phased-array surface coil. The acquisition protocol included T2-weighted imaging (T2W), diffusion-weighted imaging (DWI), and dynamic contrast-enhanced (DCE) imaging, according to the guidelines of the European Society of Urogenital Radiology [26]. Experienced radiologists analyzed images at each institution and reported using PI-RADS v2.0 until 2019 [27] and v2.1 later [5].

Men selected for prostate biopsy were those with PI-RADS score ≥ 3, and those with PI-RADS < 3 and high risk of sPCa due to a suspicious DRE, PCa family history, or a PSA density above 0.15 ng/mL^2^. Prostate biopsies were conducted in each participant center by experienced operators. Two- to four-core MRI-transrectal ultrasound cognitive or software image fusion targeted biopsies of suspicious lesions and 12-core systematic biopsy were conducted in men with PI-RADS ≥ 3 [28]. Men with PI-RADS < 3 underwent only 12-core systematic biopsy. Prostate biopsies were conducted via transrectal route in 3760 cases (75.1%) and via transperineal route in 1245 (24.9%) [29].

The prostate biopsy material was analyzed by experienced pathologists in each pathology department. PCa was reported according to the International Society of Urologic Pathology (ISUP) grade group classification. Cases were classified as sPCa when the grade group was 2 or higher [30].

### 2.3. Predictive Variables Included in the Models and Outcome Variable

Anonymized datasets, following the standards of reporting for MRI-targeted biopsy studies (START) of the prostate, were provided by each participant center for harmonization and analysis at the coordinator center [31]. Predictive variables were recorded during the PCa diagnosis approach. These variables were age (years); PCa family history (no vs. yes); type of prostate biopsy (repeated vs. initial); serum PSA level (ng/mL); and DRE (normal vs. suspicious). Additionally, prostate volume (mL), and PI-RADS score (1 to 5) were extracted from MRI images. The outcome variable was sPCa (yes vs. no).

### 2.4. Algorithms Used for Model Development

Two models were built to assess which strategy would offer the best sPCa prediction. The BCN predictive model [12] consisted of a logistic regression where sPCa was the outcome variable and seven clinical variables were included. The GMV predictive model, used a FNN SimpleNet architecture, including the same clinical variables [18]. SimpleNet features a lightweight design, making it ideal for efficient computation and rapid experimentation. The GMV model architecture consisted of three layers (fc), two with ReLU activation functions, and the third one with a sigmoid output function. The model was implemented using the PyTorch version 2.5.0 deep learning framework, with 32 neurons in the first and second layers and 16 neurons in the third layer. The binary cross-entropy loss (BCELoss) function was selected as the optimal loss function, while the RMSprop optimizer was employed for parameter optimization, maintaining a fixed learning rate of 0.001. The training set used the full training set, employing mini-batch gradient descent with a batch size of 32, over the course of 50 epochs.

**Figure A1:**
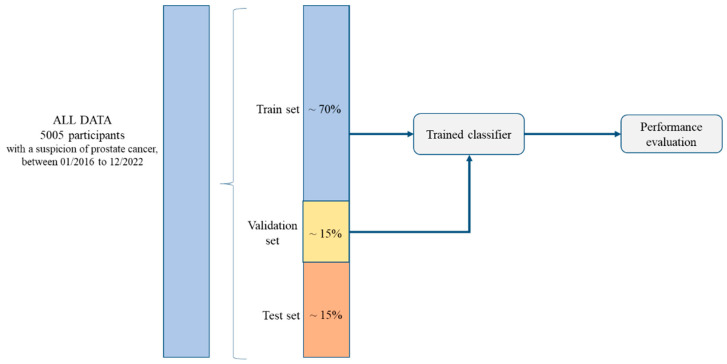
Diagram of dataset split and participant distribution across model development datasets. This diagram provides a detailed overview of the dataset division and participant allocation across machine learning model development stages. The dataset is split into three subsets: 85% for training and 15% for testing. A total of 15% of the training set was selected for validation. The training subset is used to develop the classifier, followed by evaluation on the validation subset to fine-tune model parameters. Finally, the trained classifier’s performance is assessed on the test subset, offering an unbiased evaluation of its generalization ability. This breakdown clarifies the distinct roles of each subset in training, internal validation, and final testing phases, ensuring a rigorous approach to model development and assessment.

Before training, several preprocessing steps were applied to the dataset to enhance model performance. First, data cleaning was conducted to handle duplicates, mismatched cases, and extreme values, ensuring that the dataset remained complete and reliable. Next, categorical variables (family history of PCa, type of biopsy, DRE, and PI-RADS) were encoded using one-hot encoding, converting them into binary vectors that were suitable for the model, and standardization was also applied to the numeric variables Age, PSA, and PV using StandardScaler 1.6.1 to ensure compatibility between features. Additionally, the dataset was split into three distinct subsets: training, validation, and test sets. This split allowed for effective model training while providing separate data for tuning hyperparameters and evaluating performance, ultimately leading to a more robust and generalizable model (Figure A1, Appendix A). Specifically, 4254 cases (85%) were allocated for training and 751 (15%) were reserved for testing. A set of 639 cases of the training set (15%) were used for validation. All data partitions retained similar values across all variables analyzed (Table A1). The binary classification task aimed to classify the cases into predefined categories of the outcome. The code for a comprehensive examination of the initial dataset and analyses can be found in the Supplementary File 00_dataset_analysis.ipynb (Supplementary Materials).

### 2.5. Statistical Analyses, Algorithm Performance, and Interpretation

Statistical analyses were conducted for the eight variables under study. Quantitative variables, expressed as means and standard deviations (SD), were compared with the Mann–Whitney test, and qualitative variables, expressed in percentages, with the Chi-square test. Odds ratios (OR) and 95% confidence intervals (CI) were assessed. The resulting SimpleNet (GMV model) was calibrated. The performance of both predictive models was compared using the metrics, true positives (TP), true negatives (TN), false positives (FP), false negatives (FN), negative predictive values (NPV), positive predictive values (PPV), specificity, sensitivity, and accuracy. Receiver operating characteristic (ROC) curves were plotted for each model to determine optimal classification thresholds. The area under the curve (AUC) and 95% CI were estimated and compared with the DeLong test. The precision–recall (PR) curve and AUC-PR in the training cohort were computed. For model interpretability, SHAP values were used to determine the feature attributions [32]. SHAP summary plots were generated to examine the model’s behavior in terms of feature importance and effects. Moreover, DCA was conducted to visualize the net benefit of each model. Both the AUCROC and net benefit were employed for model discrimination analysis. The clinical effectiveness of each model was assessed through the clinical utility curve (CUC), representing the rates of avoided prostate biopsies and the undetected rate of sPCa according to the continuous probability threshold. GMV and BCN models were developed using the Python programming language v. 3.10.12 (Python Software Foundation, Scotts Valley, CA, USA). Statistical analyses were conducted using the R programming v.4.3.3 language (R Foundation for Statistical Computing, Viena, Austria), and SPSS v.29 (IBM, statistical package for social sciences, San Francisco, CA, USA).

## 3. Results

### 3.1. Participant Characteristics

A total of 5005 men suspected of having PCa were included in this study. Among these, 4790 (95.7%) had a serum PSA above 3.0 ng/mL, and only 215 (4.3%) had a suspicious DRE. All participants who underwent multiparametric MRI (mpMRI) as well as targeted and/or systematic biopsies were included in the study. Participant characteristics for the overall study population are presented in Table 1. Overall, 2097 participants (41.9%) were diagnosed with sPCa, whereas 2908 (58.1%) were found to have non-signi-ficant PCa (nsPCa), a category that included both individuals without PCa and those with iPCa. The average age at the time of prostate biopsy was significantly higher in participants with sPCa than in those with nsPCa. Additionally, the mean serum PSA level was 20 ng/mL in the sPCa group compared to 8.4 ng/mL in the nsPCa group, while the mean prostate volume was 51.9 mL in the sPCa group versus 69.1 mL in the nsPCa group. Regarding PI-RADS scores, the sPCa group had higher percentages of scores 4 (47.3%) and 5 (39%), whilst the nsPCa predominantly had scores 3 (35.9%) and 4 (35.2%). Based on the computed *p*-values, no significant differences were observed among the variables across the training, validation, and test sets, indicating a homogeneous data split (Table A1).

### 3.2. Model Performance, Calibration, and Validation of the GMV and BCN Predictive Models

The key factors influencing the performance of the GMV predictive model were evaluated. Class imbalance was acknowledged as a potential concern in this analysis; however, after conducting balancing tests, it was determined that adjustments were not necessary. The models demonstrated robust performance across all classes, indicating that the existing distribution did not significantly impact the results. Additionally, the evaluation metrics used provided sufficient insight into model effectiveness.

Given the moderate size of the training set, a simple network with two hidden layers was chosen to balance model capacity and avoid overfitting, as more complex architectures did not yield significant performance improvements and increased computational complexity. The optimal network architecture was determined using hyperparameter optimization with the OPTUNA library, testing various configurations for the number of neurons, loss function, optimizer, and learning rate. The best-performing model achieved an objective value of 12.36 (Trial 63) with a configuration of hidden_size = 32, hidden_size_2 = 16, RMSprop optimizer, and a learning rate of 0.00102.

Despite some variation in other trials, with performance differences ranging from 13.53 to 16.27, the model showed relatively consistent performance, suggesting stability and robustness across different hyperparameter settings. These results indicate that the model is not overly sensitive to minor changes in hyperparameters, minimizing the risk of overfitting.

**Figure 1:**
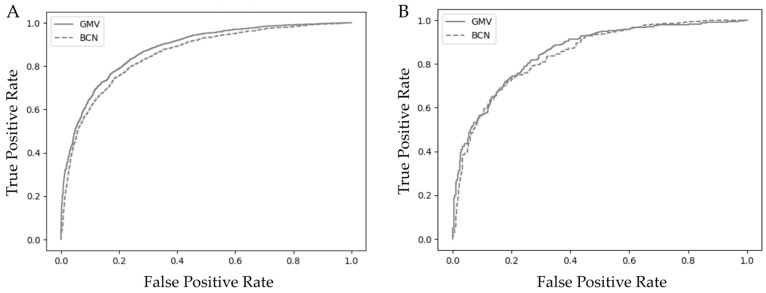
ROC curves comparing the discrimination ability for sPCa of the GMV and BCN predictive models in the training cohort (**A**) and the test cohort (**B**).

**Figure A2:**
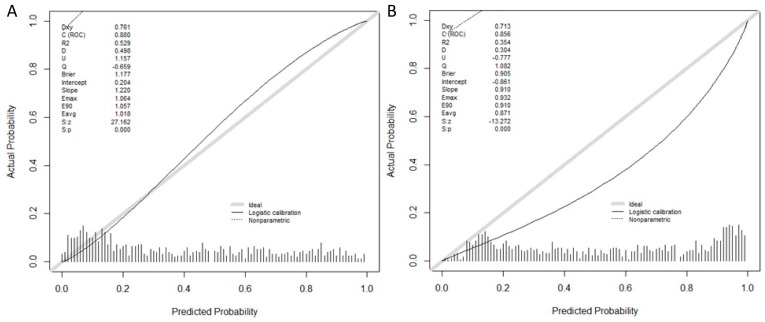
(**A**,**B**) Calibration plots for the BCN and GMV predictive models on training dataset. Predicted probabilities against observed outcomes for each model on the training dataset. The calibration curves assess each model’s ability to predict probabilities that reflect true clinical outcomes, where closer alignment of the curve to the diagonal line indicates better calibration and more accurate probability estimation. These plots offer insights into the consistency and reliability of each model’s predictions across the probability range.

Calibration of the BCN and GMV models showed that the BCN model slightly overestimated predictions at higher probability thresholds, whereas the GMV model moderately underestimated predictions in the middle range of probability thresholds (Figure A2). In the training cohort, the area under the receiver operating characteristic (ROC) curve (AUC) was 0.88 (95% CI, 0.87–0.90) for the GMV model and 0.85 (95% CI, 0.84–0.86), for de BCN model, *p* > 0.05 (Figure 1A). In the test cohort, the AUC was 0.85 (95% CI, 0.83–0.88) for the GMV model and 0.84 (95% CI, 0.82–0.86) for the BCN model, *p* > 0.05 (Figure 1B).

**Figure A3:**
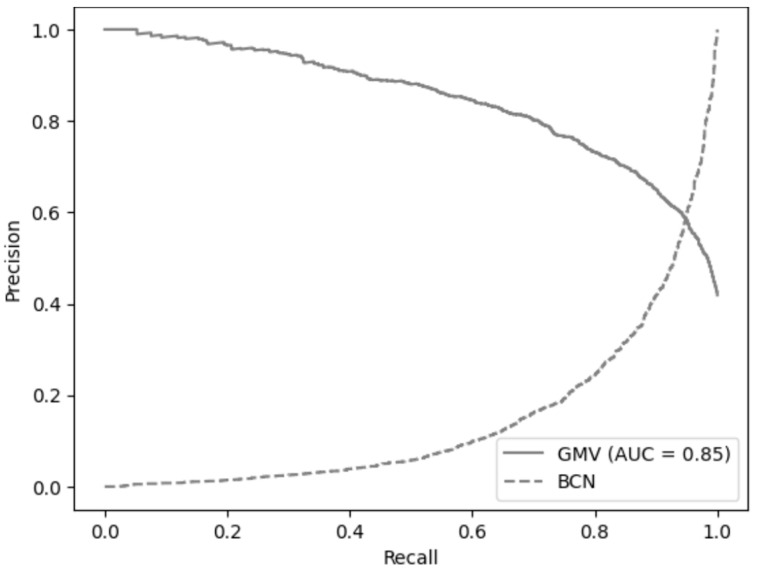
Precision–recall curves assessing model performance.

The precision–recall (PR) curve and AUC-PR in the training cohort were computed to further assess model performance. As shown in Figure A3 the PR curve is consistent with the ROC analysis, and the AUC-PR of 0.85 (95% CI, 0.82–0.87) supports the model’s robustness.

Comparative performance metrics of the GMV and BCN predictive models across the training, validation, and test datasets are summarized in Table 2. The validation dataset comprised 631 cases (15% of the training dataset). The metrics include AUC, precision, recall, F1 score, accuracy, sensitivity, specificity, the Kappa index, and Matthew’s correlation coefficient (MCC), providing a comprehensive evaluation of each model’s predictive capabilities. This consolidated format allows for a clear comparison of model performance across different datasets, highlighting the strengths and consistency of each model on unseen data.

The GMV model outperformed the BCN model across both the training and validation datasets. Specifically, the GMV model achieved AUC values of 0.88 for both training and validation datasets, while the BCN model exhibited slightly lower AUC values of 0.85 and 0.86, respectively. In the test dataset, both models experienced a slight decline in AUC; however, the GMV model still outperformed the BCN model, with an AUC of 0.85 versus 0.84. Nonetheless, these differences were not statistically significant, *p* > 0.05. Regarding precision, the BCN model demonstrated a slight advantage over the GMV model in both the training and test stages. The BCN model achieved precision scores of 0.7435 in the training dataset and 0.7607 in the test dataset, compared to the GMV model’s of 0.7171 and 0.7126, respectively. This suggests that the BCN model is marginally more accurate when making positive predictions, with a higher likelihood of correctly identifying true positives. In contrast, recall values favored the GMV model across all datasets. Specifically, the GMV model achieved recall values of 0.8266 in the training dataset, 0.8284 in the validation dataset, and 0.7556 in the test dataset, compared to the BCN model’s recall values of 0.7435, 0.7537, and 0.6762, respectively. These results indicate that the GMV model was more effective at capturing true positive cases, suggesting it may be better at identifying individuals who truly have sPCa.

In terms of specificity, the BCN model outperformed the GMV model, particularly in the test dataset, where the BCN model achieved a specificity of 0.8463 compared to GMV’s 0.7798. This indicates that the BCN model was better at correctly identifying negative cases and minimizing false positives. Both models showed similar accuracy rates, with the GMV model slightly ahead in the training and validation phases (0.7908 and 0.7919, respectively) compared to the BCN model (0.7851 and 0.7872, respectively); however, the difference in accuracy was negligible in the test dataset. The F1 score, which balances precision and recall, also favored the GMV model across all datasets. The GMV achieved F1 scores of 0.768 in training, 0.7695 in validation, and 0.7334 in testing, while the BCN model obtained scores of 0.7435, 0.7481, and 0.716, respectively. This further supports that the GMV model maintained a better balance between precision and recall than the BCN model. Regarding the Kappa score, both models demonstrated moderate agreement beyond chance, indicating that the models’ classifications were not entirely due to random chance. The MCC, which reflects the quality of binary classifications, also consistently favored the GMV model, suggesting its overall classification performance was superior to that of the BCN model.

Overall, the GMV model demonstrated superior performance across most performance metrics, particularly in recall and F1 score. This suggests that the GMV model is better at capturing true positives, which is critical in applications where identifying all potential positive cases is a priority. In terms of ROC and AUC, the GMV model exhibited a better balance between sensitivity (recall) and specificity, given its higher AUC in training and better overall performance in testing. Although both models showed similar AUC results in the test dataset, the GMV model outperformed the BCN model in terms of AUC during training and demonstrated a more favorable ROC curve, suggesting more consistent and robust model performance across datasets.

### 3.3. Variable Importance Interpretation with SHapley Additive exPlanations (SHAP)

**Figure A4:**
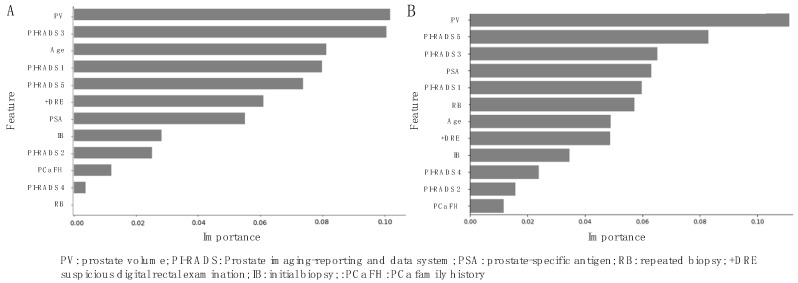
Bar plots for feature importance in prediction for GMV and BCN predictive models. Bar plot (**A**) corresponds to the GMV predictive model, and (**B**) to the BCN predictive model. The bar plots use mean absolute SHAP values to measure each feature’s contribution to the model’s predictions. The x-axis corresponds to the individual features used in the model, while the y-axis displays the mean absolute SHAP value for each feature. A higher value on the y-axis indicates a greater contribution to the model’s prediction, with features associated with longer bars being more influential in the model’s decision-making process. This visualization highlights the key variables that drive the predictions of each model, helping to identify which features play the most significant role in determining outcomes. The comparison between the two models reveals how the importance of various features differs, shedding light on the factors each model relies on to classify the data.

SHAP values are a concept used to explain the output of machine learning models. They are based on cooperative game theory, specifically the Shapley value, which provides a fair method for distributing a total gain (or outcome) among different players based on their contributions. Global SHAP values were used to assess the overall importance of individual features across the entire dataset. The distribution of SHAP values for each feature across all data points (Figure A4) revealed that features such as prostate volume and PI-RADS 5 had the highest SHAP values, indicating their significant impact on the GMV model’s output (Figure A4A). PSA and PI-RADS 3 also had substantial influence, whereas lower-ranked features, such as family history of PCa and repeated prostate biopsy, contributed minimally to the prediction. For the BCN model (Figure A4B), the two most important features were prostate volume and PI-RADS 3, both with SHAP values around 0.1, suggesting they had nearly equal influence on model predictions. Age and PI-RADS 1 also contributed significantly, though to a lesser extent. Features such as PCa family history, PI-RADS 4, and repeated biopsies had minimal to negligible SHAP values, making them less relevant in this context. Both models consistently identified prostate volume as the most decisive feature in predicting sPCa outcomes, while they also agreed that repeated biopsies were the least relevant factor.

**Figure 2:**
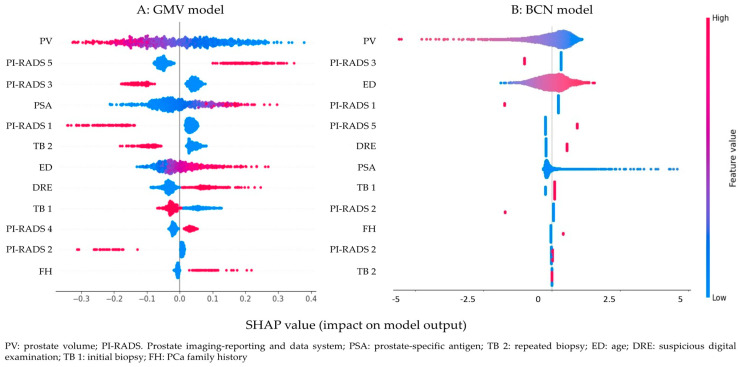
Beeswarm plot illustrating the contribution of variables to the prediction of significant prostate cancer (sPCa) for the GMV predictive model (**A**) and the BCN predictive model (**B**).

The SHAP Beeswarm plot (Figure 2) provided valuable insights into the features that most influenced the model’s predictions, offering a clearer understanding of model behavior and supporting more informed decision-making. Each point represents a single prediction’s SHAP value of a single prediction, with vertical jittering applied to enhance visibility. The color gradient indicates the feature’s value, ranging from low (blue) to high (red). This plot highlights both the direction (positive or negative impact) and the distribution of SHAP values, revealing patterns and interactions that contribute to the model’s predictions. In both models, high values of prostate volume (shown in red) demonstrated an inverse relationship with sPCa risk, indicating that larger prostate volume was associated with a lower likelihood of sPCa, whereas smaller prostate volumes increased the risk (Figure 2A,B).

A similar pattern was observed for PI-RADS 1, 2, 3, and repeated biopsies. In contrast, higher values of PI-RADS 4, 5, age, and suspicious digital rectal examination (also shown in red) were associated with a greater risk of sPCa, reflecting their positive contribution to the outcome. Interestingly, these models exhibited different directionality regarding initial biopsies. In the GMV model, undergoing an initial biopsy was associated with a lower probability of a positive sPCa outcome, whereas the BCN model showed the opposite trend. This discrepancy suggests that the relationship between initial biopsy status and sPCa may not be straightforward and could differ based on model-specific feature interactions.

### 3.4. Clinical Comparison of GMV and BCN Predictive Models for sPCa Detection

The net benefit of the GMV and BCN predictive models was analyzed with decision curve analysis (DCA). In this analysis, the x-axis represents the predicted probabilities, while the y-axis indicates the proportion of actual positive cases identified by the model. The blue line corresponds to the GMV model, and the orange line represents the BCN model. For reference, the gray line represents the “Biopsy None” strategy, in which no instances are classified as positive, resulting in unidentified positive cases and potentially missing all at-risk patients. Conversely, the green line represents the “Biopsy All” strategy, where all instances are classified as positive, ensuring that all positive cases are identified but also leading to a high number of false positives, which may result in unnecessary prostate biopsies. DCA provides a comparative framework for assessing model performance relative to these two extreme strategies, illustrating the trade-offs between sensitivity and specificity at different threshold probabilities.

**Figure 3:**
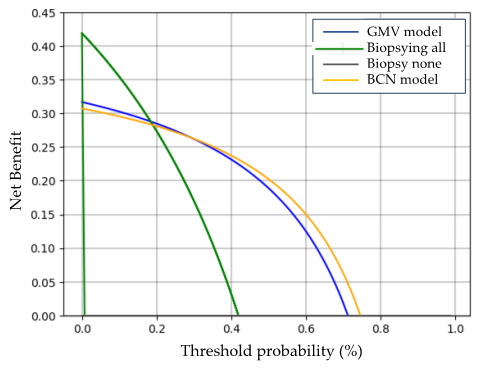
Net benefit of the GMV and BCN predictive models compared to the “biopsy all” and “biopsy none” strategies, as assessed by Decision Curve Analysis (DCA).

The BCN predictive model demonstrated a higher net benefit than the GMV model across a wide range of threshold probabilities. DCA indicated that the GMV model provided a higher net benefit for threshold probabilities between 1% and 35%, whereas the BCN model outperformed it at threshold probabilities ranging from 36% to 75% (Figure 3). This suggests that the BCN model may be slightly more effective at identifying high-risk patients. However, both models performed similarly within the critical range of 10% to 35%, where most clinical decisions are made. As threshold probabilities for sPCa detection increased beyond 30%, both models deviated further from the performance of the “Biopsy All” strategy, reinforcing their clinical utility. Additionally, both models consistently maintained a gap above the “Biopsy All” and “Biopsy None” curves across various thresholds, highlighting their overall robustness and clinical applicability.

**Figure 4:**
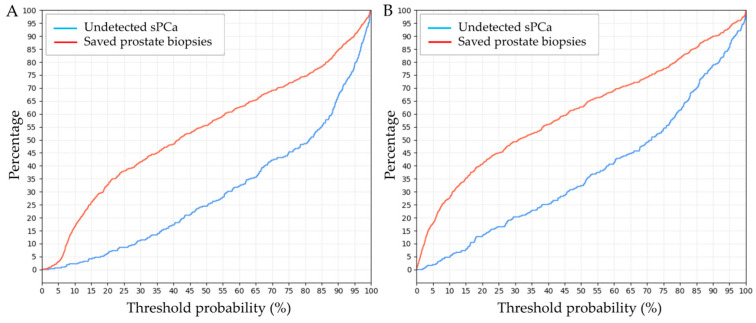
Clinical utility curves (CUC) for the GMV model (**A**) and the BCN model (**B**). The x-axis represents varying threshold probability points (expressed as percentages), indicating the probability above which a prostate biopsy is recommended. The y-axis displays two key metrics, the rate of saved biopsies (red line) and the rate of undetected sPCa (blue line), as the continuous threshold probability for sPCa prediction increases continuously.

Clinical utility curves (CUC) illustrate the rates of saved prostate biopsies and undetected sPCa as the continuous threshold probability for sPCa prediction continuously increases in both models (Figure 4).

The GMV model demonstrated a 23% reduction in prostate biopsies at a 14% probability threshold for sPCa, with only 2.6% of sPCa cases remaining undetected. Even at a higher 20% threshold, it maintained a substantial 32.5% biopsy reduction while missing 6.5% of sPCa cases. In comparison, the BCN model achieved greater biopsy savings but at the cost of higher missed sPCa rates. At a 14% threshold, the BCN model reduced prostate biopsies by 34%, but 8% of sPCa cases remained undetected. At a 20% threshold, biopsy savings increased to 41%, though the missed sPCa rate also rose to 13%. The rates of avoided prostate biopsies and undetected sPCa for the GMV and BCN predictive models at thresholds ranging from 5% to 20% are summarized in Table 3. When fixing a clinically appropriate 95% sensitivity, the GMV model avoided between 27.5% and 29% of prostate biopsies at thresholds between 16% and 17%. In contrast, the BCN model avoided between 27% and 27.5% of prostate biopsies at lower thresholds of 9% to 10%.

## 4. Discussion

This study compared the clinical performance of two predictive models for sPCa detection in prostate biopsies, both developed using seven clinical variables obtained during the diagnostic process. The GMV model, based on a SimpleNet-FNN-based ML algorithm, demonstrated clinical effectiveness comparable to that of the BCN model, which was developed using a classic LR approach. Both models demonstrated appropriate calibration and strong performance, though each had distinct advantages depending on clinical priorities. The GMV model exhibited a discrimination ability with an AUC of 0.85, comparable to the 0.84 achieved by the BCN model [33,34,35,36,37,38,39,40,41,42]. The GMV model prioritized recall, making it particularly suitable for scenarios where detecting men with sPCa and minimizing false-negative predictions is critical, such as in cancer diagnosis. In contrast, the BCN model showed higher precision and specificity, making it more effective in reducing false-positive predictions and unnecessary prostate biopsies.

The use of predictive models offers a significant advantage over the use of biomarkers in improving the current diagnostic approach for sPCa, as they are often expensive and require the collection of biological fluids for analysis [10,11]. However, ensuring the availability of a freely accessible risk calculator web-based or smartphone application would be very beneficial for clinical practice as patients could be efficiently classified in the early stages of diagnosis. This is exemplified by the BCN risk calculator, which is available at https://mripcaprediction.shinyapps.io/MRIPCaPrediction/ (accessed on 12 February 2025) [12]. Regarding net benefit analysis, the GMV model prioritized minimizing missed sPCa detections while achieving moderate reductions in prostate biopsies. In contrast, the BCN model focused on maximizing biopsy reduction, albeit at the cost of a higher number of missed sPCa cases. Nonetheless, the clinically optimal threshold detecting 95% of sPCa of the BCN model provided a balance similar to that of the GMV model, aligning with the performance of comparable models. The GMV model, designed using the SimpleNet-FNN framework, offers modularity for future integration of diverse data sets, including genomics and other omics such as MRI radiomics, enhancing its adaptability. While the BCN model is similarly effective, it is less expandable [43,44,45].

The choice between these models for clinical implementation should align with specific clinical priorities. If minimizing missed diagnoses (false negatives) and maximizing sPCa detection is the primary concern, the GMV model is preferable. Conversely, if reducing unnecessary prostate biopsies and false positives is the priority, the BCN model would be the better option. The optimal threshold for clinical decision-making with the GMV model was found to be 14%, at which it could reduce prostate biopsies by 23% while missing only 2.6% of sPCa. Even at a higher threshold of 20%, the GMV model could avoid 32.5% of biopsies, with an acceptable trade-off of 6.5% missed sPCa. In comparison, at a threshold of 14%, the BCN model avoided 34% of biopsies but at the cost of an 8% rate of undetected sPCa. At a threshold of 20%, the BCN model saved 41% of biopsies, with a corresponding 13% rate of undetected sPCa cases. The ideal threshold for the BCN model appeared to be around 13%, allowing a 33.5% reduction in biopsies while maintaining a 6.5% rate of missed reclassifications. The BCN model performed similarly to other previously reported models, which, at a 20% threshold, reduced biopsies by 42% but had a 7% rate of missed cancers [46]. This suggests that the GMV model prioritizes minimizing missed sPCa detections, even at the cost of performing more biopsies. However, when both models were compared at a fixed 95% sensitivity, the GMV model achieved a slightly higher percentage of avoidable prostate biopsies.

SHAP analysis identified prostate volume as the most influential feature in the GMV model, with higher prostate volume values inversely associated with the likelihood of sPCa. This aligns with the traditional analysis of independent predictive variable weights in LR models [12,13,14]. Additionally, a PI-RADS score of 5 was a key determinant in the GMV model, whereas a PI-RADS score of 3 and age played significant roles in the BCN model, consistent with previous findings [18]. A discrepancy in the SHAP analysis was observed in the effect of the initial biopsy feature, which had differing impacts between the models. In the GMV model, initial biopsy status was associated with a lower probability of sPCa, as previously reported. However, in the BCN model, initial biopsy status did not demonstrate a significant relationship with sPCa detection, suggesting that it may not be a relevant predictor in this model. This difference can be attributed to the difference in the weights of predictive variables regarding the model. Other risk factors, such as DRE, exhibited moderate importance in both models. Conversely, PCa family history did not emerge as a highly relevant factor in either predictive model, despite being a well-established risk factor for sPCa. Previous studies have identified its predictive significance [12,47], but those models did not incorporate PI-RADS scores, which may have altered the relative weight and ranking of predictive variables. Additionally, the evolving demographic profile of PCa diagnoses may suggest that a growing proportion of cases occur in men without a known family history of the disease. Nevertheless, PCa family history remains an important risk factor and should not be excluded from predictive models or preventive medicine strategies. Overall, the SHAP analysis provided valuable insights into the clinical features driving model predictions, enhancing the interpretability of both the GMV and BCN models. These findings, in combination with clinical threshold evaluations, offer a clearer understanding of how these models can be effectively applied in clinical practice to support informed decision-making in sPCa detection.

Several limitations of our study must be acknowledged. Its retrospective nature may have introduced selection bias, as the included population was determined by the availability of pre-existing data. The analyzed cohort was derived from an opportunistic PCa screening program, which may have introduced biases in participant characteristics compared to those identified through population-based screening programs. While our study included a substantial number of men suspected of having PCa, the limited sample size for specific subgroup analyses may have reduced the statistical power and robustness of these analyses, potentially affecting the reliability of our conclusions. As a multicenter study without centralized diagnostic procedures, some degree of variability was likely inevitable, even with well-established criteria for identifying men with suspected PCa, performing and interpreting MRI scans, and conducting and reporting prostate biopsies. Variations in expertise across centers may have further contributed to this variability. The current definition of sPCa in prostate biopsies does not correlate well with the true pathology of the entire prostate gland, which can be only assessed when radical prostatectomy is performed as a local treatment for the tumor [48].

After this analysis, we recognize that both machine learning and logistic regression algorithms resulted in small differences when developing predictive models for sPCa based on seven clinical variables, which represents a small dataset. These differences produced minor changes in the clinical performance metrics of both models. However, their effectiveness was nearly identical at the 95% sensitivity threshold for sPCa detection, which is highly appropriate for this purpose. The GMV model requires a web-based or smartphone application for routine clinical use [49]. Moreover, the GMV model offers greater potential for integration into federated networks, as its online evolution could be supported through continuous model updates [16]. Additionally, the GMV model can incorporate other data types such as genomic, proteomic, or radiomic features from MRI, enabling the processing of large data sets, an approach not supported by logistic regression models [50,51].

## 5. Conclusions

Both ML and LR models achieve high accuracy in predicting sPCa, with ML favoring recall and LR optimizing specificity. Future studies should explore external validation and the integration of multimodal data to refine predictive accuracy.

## Figures and Tables

**Table 1:** Characteristics of participants and odds ratio estimates for clinical variables in a logistic regression analysis for significant prostate cancer.

Characteristic	sPCa	nsPCa	Odds Ratio (95% CI)	*p*-Value
Number of men (%)	2097 (41.9)	2908 (58.1)	-	-
Mean age, years (SD)	70 (8.2)	66 (7.6)	1.07 (1.06–1.08)	<0.001
Mean serum PSA, ng/mL (SD)	20 (109)	8.4 (9.6)	1.04 (1.03–1.05)	<0.001
PCa family history, *n* (%)				
No	1930 (92%)	2723 (93.6%)	-	Ref.
Yes	167 (8%)	185 (6.4%)	1.27 (1.02–1.58)	0.033
Type of prostate biopsy, *n* (%)				
Initial	1594 (76%)	1906 (65.5%)	-	Ref.
Repeated	503 (24%)	1002 (34.5%)	0.6 (0.53–0.68)	<0.001
DRE, *n* (%)				
Normal	1161 (55.4%)	2417 (83.1%)	-	Ref.
Suspicious	936 (44.6%)	491 (16.9%)	3.97 (3.49–4.52)	<0.001
Prostate volume (mL)	51.9 (27.6)	69.1 (34.4)	0.98 (0.98–0.98)	<0.001
PI-RADS score, *n* (%)				
1	60 (2.9%)	514 (17.7%)	-	Ref.
2	23 (1.1%)	152 (5.2%)	1.3 (0.76–2.14)	0.322
3	206 (9.8%)	1044 (35.9%)	1.69 (1.25–2.31)	0.001
4	991 (47.3%)	1024 (35.2%)	8.29 (6.31–11.08)	<0.001
5	817 (39%)	174 (6%)	40.22 (29.61–55.48)	<0.001

CI: confidence interval; SD: standard deviation; PCa: prostate cancer; sPCa: significant PCa; nsPCa: non-significant PCa; PSA: prostate-specific antigen; DRE: digital rectal examination; PI-RADS: Prostate Imaging-Reporting and Data System.

**Table 2:** Comparative performance metrics for the GMV and BCN predictive models across training, validation, and test datasets with key values for each metric.

Metric	Training Set (*n* = 4–254)		Validation Set (*n =* 631)		Test Set (*n* = 751)	
	GMV Model	BCN Model	GMV Model	BCN Model	GMV Model	BCN Model
AUC (95% CI)	0.88 (0.87−0.90)	0.85 (0.84−0.86)	0.88 (0.86−0.91)	0.86 (0.85−0.87)	0.85 (0.83−0.88)	0.84 (0.82−0.86)
Precision (95% CI)	0.7171 (0.6973−0.7362)	0.7435 (0.7228−0.7633)	0.7184 (0.6659−0.7657)	0.7426 (0.6876−0.7910)	0.7126 (0.6618−0.7585)	0.7607 (0.7074−0.8069)
Recall (95% CI)	0.8266 (0.8083−0.8435)	0.7435 (0.7228−0.7633)	0.8284 (0.7787−0.8688)	0.7537 (0.6988−0.8015)	0.7556 (0.7052−0.7997)	0.6762 (0.6227−0.7255)
Specificity (95% CI)	0.765 (0.7478−0.7813)	0.8151 (0.7993−0.8299)	0.7655 (0.7198−0.8058)	0.8113 (0.7684−0.8479)	0.7798 (0.7386−0.8162)	0.8463 (0.8095−0.8771)
Accuracy (95% CI)	0.7908 (0.7783−0.8027)	0.7851 (0.7725−0.7972)	0.7919 (0.7587−0.8215)	0.7872 (0.7538−0.8171)	0.7696 (0.7382−0.7983)	0.7750 (0.7437−0.8034)
F1 score (95% CI)	0.768 (0.7537−0.7832)	0.7435 (0.7275−0.7592)	0.7695 (0.7298−0.8047)	0.7481 (0.7025−0.7842)	0.7334 (0.6952−0.7695)	0.7160 (0.6736−0.7537)
Kappa score (95% CI)	0.5792 (0.5548−0.6037)	0.5587 (0.5325−0.5847)	0.5815 (0.5163−0.6389)	0.5639 (0.4903−0.6276)	0.5309 (0.4671−0.5851)	0.5307 (0.4671−0.5911)
MCC (95% CI)	0.5841 (0.5603−0.6085)	0.5587 (0.5325−0.5850)	0.5864 (0.5243−0.6441)	0.5639 (0.4910−0.6287)	0.5316 (0.4682−0.5857)	0.5332 (0.4685−0.5948)

AUC: area under the curve; MCC: Matthew’s correlation coefficient; CI: confidence interval.

**Table 3:** Rates of avoided prostate biopsies and undetected sPCa provided with the GMV and BCN models at threshold probabilities between 5% and 20%.

Threshold (%)	GMV Model		BCN Model	
	Saved Biopsies (%)	Undetected sPCa (%)	Saved Biopsies (%)	Undetected sPCa (%)
5	3.5	0.5	18	2
6	5	0.75	20	2.5
7	9.5	1	23	3.5
8	10	2	26	4.75
9	14	2	27	5
10	17	2	27.5	5
11	19	2.5	30	6
12	20	2.5	32.5	6.5
13	22	2.6	33.5	6.5
14	23	2.6	34	8
15	26	4.5	35	8.5
16	27.5	5	36	10.5
17	29	5	37.5	10.5
18	30	5.1	39.5	13
19	30	5.1	40	13
20	32.5	6.5	41	13

## Data Availability

Data are available under request to the corresponding author.

## Supplementary Materials

The following supporting information can be downloaded at: https://www.mdpi.com/article/10.3390/cancers17071101/s1, S1: Dataset. S2. Metrics. S3. Model Training.

## Appendix A

**Table A1:** Demographic, clinical, and imaging characteristics of the training, validation, and test datasets.

Characteristic	Development Cohort	Validation Cohort	Test Cohort	*p*-Value
Number of men	4254	639	751	
Mean age at biopsy, years (SD)	68 (8.3)	68 (8.3)	68 (8.2)	1
Mean serum PSA, ng/mL (SD)	13.4 (75.0)	12.7 (35.5)	12.8 (47.6)	0.36
Suspicious DRE, *n* (%)	1210 (28.5)	192 (30.1)	217 (28.9)	0.11
PCa family history, *n* (%)	304 (7.2)	51 (8.0)	48 (6.4)	0.2
Mean prostate volume, mL (SD)	61.5 (32.3)	62.0 (33.1)	64.6 (35.8)	0.65
Previous negative prostate biopsy, *n* (%)	1281 (30.2)	216 (33.9)	224 (29.9)	0.41
PI-RADS version used	2	2.1	2	
Mean number of suspicious lesions	2	2	2	
PI-RADS score of index lesion, *n* (%)				
1	470 (11.1)	71 (11.2)	104 (13.9)	0.33
2	148 (3.5)	15 (2.4)	27 (3.6)	0.09
3	1053 (24.8)	161 (25.2)	197 (26.3)	0.27
4	1743 (41.0)	261 (40.9)	272 (36.3)	0.07
5	840 (19.8)	131 (20.6)	151 (20.2)	0.71
sPCa detection, *n* (%)	1782 (41.9)	268 (42.0)	315 (42.0)	1

SD: standard deviation; PSA: prostatic specific antigen; DRE: digital rectal examination; PI-RADS: Prostate Imaging-Reporting and Data System; PCa: prostate cancer; sPCa: significant PCa.
