# Supplementary material for: Developing a Predictive Model for Significant Prostate Cancer Detection in Prostatic Biopsies from Seven Clinical Variables: Is Machine Learning Superior to Logistic Regression?
Source: Cancers (Basel). 2025 Mar 25;17(7):1101. doi: 10.3390/cancers17071101 (PMC11987821; doi:10.3390/cancers17071101)
Supplement: Supplementary file 1 [file cancers-17-01101-s001.zip › cancers-3521745-supplementary.pdf]

S1. Dataset

```python
import torch
import numpy as np
import pandas as pd

from sklearn.model_selection import train_test_split
from sklearn.preprocessing import StandardScaler
from sklearn.compose import ColumnTransformer
from torch.utils.data import TensorDataset, DataLoader

def get_dataset(number_records=False):

    #########################
    # Dataset preprocessing #
    #########################

    clinical_data = pd.read_csv("dataset/Vhir_5005_240124.csv", sep=",")
    clinical_data.rename(columns={"PV":"VP", "SCP":"csPC"}, inplace=True)
    clinical_data.drop(columns=["Unnamed: 0", "SER"], inplace=True)

    if number_records:
        print("Number of positive and negative records in dataset: ",
clinical_data.groupby(clinical_data["csPC"])["csPC"].count(), "\n")

    ###########################
    # Variables preprocessing #
    ###########################

    clinical_data_pir = pd.get_dummies(clinical_data.PIR, prefix='PIR',
dtype=int)
    clinical_data_tb = pd.get_dummies(clinical_data.TB, prefix='TB',
dtype=int)

    clinical_data = pd.concat([clinical_data, clinical_data_pir,
clinical_data_tb], axis=1)
    clinical_data.drop(columns=["PIR", "TB"], inplace=True)

    return clinical_data

def train_test_validation_split(number_records=False):

    clinical_data = get_dataset(number_records)

    ######################################
    # Split the data into train and test #
    ######################################
```

```
    # Inclusion of stratify=y to obtain the same proportion of target
categories in both train_model and test
    seed = 42
    train, test = train_test_split(clinical_data, test_size=0.15,
random_state=seed, stratify=clinical_data["csPC"])
    _, validation = train_test_split(train, test_size=0.15,
random_state=seed, stratify=train["csPC"])

    if number_records:
        print("Number of positive and negative records in train dataset:
", train.groupby(train["csPC"])["csPC"].count(), "\n")
        print("Number of positive and negative records in test dataset:
", test.groupby(test["csPC"])["csPC"].count(), "\n")
        print("Number of positive and negative records in validation
dataset: ", validation.groupby(validation["csPC"])["csPC"].count(), "\n")

    ##########################################################
    # Split the data into BCN results and clinical variables #
    ##########################################################

    train_BSC = train[["BCN2", "csPC"]]
    test_BSC = test[["BCN2", "csPC"]]
    validation_BSC = validation[["BCN2", "csPC"]]

    train = train.drop(columns=["CP", "BCN2"])
    test = test.drop(columns=["CP", "BCN2"])
    validation = validation.drop(columns=["CP", "BCN2"])

    return train_BSC, test_BSC, validation_BSC, train, test, validation

def standarization(number_records=False):

    train_BSC, test_BSC, validation_BSC, train, test, validation =
train_test_validation_split(number_records)

    ############################
    # Variables standarization #
    ############################

    sc = ColumnTransformer([('standar_scaler', StandardScaler(), ['ED',
'PSA', 'VP'])], remainder='passthrough')

    columns = ['ED', 'PSA', 'VP', 'AF', 'TR', 'csPC', 'PIR_1', 'PIR_2',
'PIR_3', 'PIR_4',
        'PIR_5', 'TB_1', 'TB_2']

    train_sc = sc.fit_transform(train)
    test_sc = sc.transform(test)
    validation_sc = sc.transform(validation)
```

```
    df_train_sc = pd.DataFrame(train_sc, columns = columns)
    df_test_sc = pd.DataFrame(test_sc, columns = columns)
    df_validation_sc = pd.DataFrame(validation_sc, columns = columns)

    ####################
    # Shuffle the data #
    ####################

    seed = 4
    np.random.seed(seed)

    # Shuffle the training data
    indices = np.arange(df_train_sc.shape[0])
    np.random.shuffle(indices)

    # Reorder the DataFrame based on shuffled indices
    df_train_sc = df_train_sc.iloc[indices]

    # Shuffle the test data
    indices = np.arange(df_test_sc.shape[0])
    np.random.shuffle(indices)

    # Reorder the DataFrame based on shuffled indices
    df_test_sc = df_test_sc.iloc[indices]

    # Shuffle the validation data
    indices = np.arange(df_validation_sc.shape[0])
    np.random.shuffle(indices)

    # Reorder the DataFrame based on shuffled indices
    df_validation_sc = df_validation_sc.iloc[indices]

    ############################################
    # Split the data into variables and target #
    ############################################

    x_train = df_train_sc[["ED", "PSA", "AF", "TR", "VP", "PIR_1",
"PIR_2", "PIR_3", "PIR_4", "PIR_5", "TB_1", "TB_2"]]
    y_train = df_train_sc[['csPC']]

    x_test = df_test_sc[["ED", "PSA", "AF", "TR", "VP", "PIR_1", "PIR_2",
"PIR_3", "PIR_4", "PIR_5", "TB_1", "TB_2"]]
    y_test = df_test_sc[['csPC']]

    x_validation = df_validation_sc[["ED", "PSA", "AF", "TR", "VP",
"PIR_1", "PIR_2", "PIR_3", "PIR_4", "PIR_5", "TB_1", "TB_2"]]
    y_validation = df_validation_sc[['csPC']]
```

```
    return train_BSC, test_BSC, validation_BSC, x_train, y_train, x_test,
y_test, x_validation, y_validation

def dataset_training_preparation(number_records=False):

    train_BSC, test_BSC, validation_BSC, x_train, y_train, x_test,
y_test, x_validation, y_validation = standarization(number_records)

    ################################
    # Preparing data for training #
    ################################

    inputs_tensor_x_train = torch.tensor(x_train.values).float()
    labels_tensor_y_train = torch.tensor(y_train.values).float()

    inputs_tensor_x_test = torch.tensor(x_test.values).float()
    labels_tensor_y_test = torch.tensor(y_test.values).float()

    inputs_tensor_x_validation =
torch.tensor(x_validation.values).float()
    labels_tensor_y_validation =
torch.tensor(y_validation.values).float()

    # Create a TensorDataset
    dataset_train = TensorDataset(inputs_tensor_x_train,
labels_tensor_y_train)
    dataset_test = TensorDataset(inputs_tensor_x_test,
labels_tensor_y_test)
    dataset_validation = TensorDataset(inputs_tensor_x_validation,
labels_tensor_y_validation)

    # Create a DataLoader
    batch_size = 32
    dataloader_train = DataLoader(dataset_train, batch_size=batch_size,
shuffle=True)
    dataloader_test = DataLoader(dataset_test, batch_size=batch_size,
shuffle=True)
    dataloader_validation = DataLoader(dataset_validation,
batch_size=batch_size, shuffle=True)

    BSC_data = {
        "train_BSC":train_BSC,
        "test_BSC":test_BSC,
        "validation_BSC":validation_BSC
    }

    data = {
        "x_train":x_train,
        "y_train":y_train,
```

```python
        "x_test":x_test,
        "y_test":y_test,
        "x_validation":x_validation,
        "y_validation":y_validation
    }

    dataloaders = {
        "dataloader_train":dataloader_train,
        "dataloader_test":dataloader_test,
        "dataloader_validation":dataloader_validation
    }

    tensors = {
        "inputs_tensor_x_train":inputs_tensor_x_train,
        "labels_tensor_y_train":labels_tensor_y_train,
        "inputs_tensor_x_test":inputs_tensor_x_test,
        "labels_tensor_y_test":labels_tensor_y_test,
        "inputs_tensor_x_validation":inputs_tensor_x_validation,
        "labels_tensor_y_validation":labels_tensor_y_validation
    }

    return BSC_data, data, dataloaders, tensors
```

S2. Metrics

```
import torch

import shap
import numpy as np
import pandas as pd
import seaborn as sns
import matplotlib.pyplot as plt
from dcurves import dca, plot_graphs
from sklearn.metrics import roc_auc_score, roc_curve, confusion_matrix
from sklearn.utils import resample
from sklearn.calibration import calibration_curve

#######
# AUC #
#######

def eval_model_auc(model, x_eval, y_eval):

    # Evaluate the model on the test data
    model.eval()
    with torch.no_grad():
        outputs = model(torch.tensor(x_eval.values).float())
        y_pred = outputs.cpu().numpy()

    # Calculate the AUC score
    auc_score = roc_auc_score(y_eval, y_pred)
    return auc_score, y_pred, y_eval

def get_auc_with_95CI(y_true, y_score):
    # Separate data into positive and negative classes
    positive_data = [(x, y) for x, y in zip(y_true.values, y_score) if x
== 1]
    negative_data = [(x, y) for x, y in zip(y_true.values, y_score) if x
== 0]

    # Calculate the AUC
    auc_observed = roc_auc_score(y_true, y_score)
    # Number of bootstrap iterations
    n_iterations = 1000

    # Initialize an array to store the bootstrapped AUC values
    auc_values = np.zeros(n_iterations)

    # Perform bootstrapping
    for i in range(n_iterations):
        # Resample with replacement separately for each class
        positive_samples = resample(positive_data)
```

```
        negative_samples = resample(negative_data)

        # Combine the samples to create a balanced dataset
        resampled_data = positive_samples + negative_samples

        # Calculate AUC on the resampled data
        auc_values[i] = roc_auc_score(*zip(*resampled_data))

    # Calculate the 95% confidence interval
    lower_bound = np.percentile(auc_values, 2.5)
    upper_bound = np.percentile(auc_values, 97.5)

    return auc_observed, lower_bound, upper_bound

##################
# Plot ROC curve #
##################

def plot_roc_curve(model, x_eval, y_eval, label_eval, fpr_BCN, tpr_BCN,
label_BCN, subset):

    # Evaluate the model on the test data
    model.eval()
    with torch.no_grad():
        outputs = model(torch.tensor(x_eval.values).float())
        y_pred = outputs.cpu().numpy()

    fpr_eval, tpr_eval, _ = roc_curve(y_eval, y_pred)

    #set up plotting area
    plt.figure(0).clf()
    plt.plot(fpr_eval, tpr_eval, label=label_eval, color='gray') # ROC
curve = TPR vs FPR
    plt.plot(fpr_BCN, tpr_BCN, linestyle='--', label=label_BCN,
color='gray')

    plt.title("ROC curve {}".format(subset))
    plt.xlabel("False Positive Rate")
    plt.ylabel("True Positive Rate")

    plt.legend()
    plt.show()


####################
# Confusion matrix #
####################

def compute_confusion_matrix(model, x_eval, y_eval):
```

```python
    model.eval()
    with torch.no_grad():
        outputs = model(torch.tensor(x_eval.values).float())
        y_pred = outputs.cpu().numpy().round()

    return confusion_matrix(y_true=y_eval, y_pred=y_pred)

def plot_confusion_matrix(cm, ax):
    classes = ('FCLASS=0', 'FCLASS=1')
    df_cm = pd.DataFrame(cm, index=classes, columns=classes)
    tot = np.sum(cm)

    sns.heatmap(df_cm, cmap='Blues', annot=True, fmt='d', ax=ax)

    # Add text annotations for counts and percentages
    for i in range(len(classes)):
        for j in range(len(classes)):
            count = df_cm.iloc[i, j]
            percentage = count / tot * 100

            text_color = 'black' if df_cm.iloc[i, j] <
np.max(df_cm.values) / 2 else 'white'

            ax.text(j + 0.5, i + 0.5, f'\n\n\n({percentage:.2f}%)',
ha='center', va='center', fontsize=11, color=text_color)

    ax.set_title('Confusion Matrix', fontsize=16)
    ax.set_xlabel('Predicted')
    ax.set_ylabel('Truth')

########################
# Sensitivity - Recall #
########################

def calculate_sensitivity(confusion_matrix):
    """
    Calculate sensitivity (recall) from the confusion matrix.

    Args:
    - confusion_matrix (torch.Tensor): Confusion matrix of size (2, 2).

    Returns:
    - sensitivity (float): Sensitivity of the model.
    """
    TP = confusion_matrix[1, 1].item()
    FN = confusion_matrix[1, 0].item()
    sensitivity = TP / (TP + FN) if (TP + FN) > 0 else 0
    return sensitivity
```

```python
###############
# Specificity #
###############

def calculate_specificity(confusion_matrix):
    """
    Calculate specificity from the confusion matrix.

    Args:
    - confusion_matrix (torch.Tensor): Confusion matrix of size (2, 2).

    Returns:
    - specificity (float): Specificity of the model.
    """
    TN = confusion_matrix[0, 0].item()
    FP = confusion_matrix[0, 1].item()
    specificity = TN / (TN + FP) if (TN + FP) > 0 else 0
    return specificity

############
# Accuracy #
############

def calculate_accuracy(confusion_matrix):
    """
    Calculate accuracy from the confusion matrix.

    Args:
    - confusion_matrix (torch.Tensor): Confusion matrix of size (2, 2).

    Returns:
    - accuracy (float): Accuracy of the model.
    """
    TP = confusion_matrix[1, 1].item()
    TN = confusion_matrix[0, 0].item()
    total = confusion_matrix.sum().item()
    accuracy = (TP + TN) / total if total > 0 else 0
    return accuracy

#############
# Precision #
#############

def calculate_precision(confusion_matrix):
    """
    Calculate precision from the confusion matrix.

    Args:
    - confusion_matrix (torch.Tensor): Confusion matrix of size (2, 2).
```

```
    Returns:
    - precision (float): Precision of the model.
    """
    TP = confusion_matrix[1, 1].item()
    FP = confusion_matrix[0, 1].item()
    precision = TP / (TP + FP) if (TP + FP) > 0 else 0
    return precision

############
# F1 score #
############

def calculate_f1_score(confusion_matrix):
    """
    Calculate the F1 score from the confusion matrix.

    Args:
    - confusion_matrix (torch.Tensor): Confusion matrix of size (2, 2).

    Returns:
    - f1_score (float): F1 score of the model.
    """
    precision = calculate_precision(confusion_matrix)
    recall = calculate_sensitivity(confusion_matrix)
    f1_score = 2 * (precision * recall) / (precision + recall) if
(precision + recall) > 0 else 0
    return f1_score

#########
# Kappa #
#########

def calculate_kappa(confusion_matrix):
    """
    Calculate Cohen's Kappa from the confusion matrix.

    Args:
    - confusion_matrix (torch.Tensor): Confusion matrix of size (2, 2).

    Returns:
    - kappa (float): Cohen's Kappa of the model.
    """
    total = confusion_matrix.sum().item()
    p_o = (confusion_matrix[0, 0] + confusion_matrix[1, 1]).item() /
total
    p_e = ((confusion_matrix[0, 0] + confusion_matrix[0, 1]).item() *
           (confusion_matrix[0, 0] + confusion_matrix[1, 0]).item() +
           (confusion_matrix[1, 0] + confusion_matrix[1, 1]).item() *
```

```
            (confusion_matrix[0, 1] + confusion_matrix[1, 1]).item()) /
(total * total)
    kappa = (p_o - p_e) / (1 - p_e) if (1 - p_e) > 0 else 0
    return kappa

#######
# MCC #
#######

def calculate_mcc(confusion_matrix):
    """
    Calculate Matthews Correlation Coefficient (MCC) from the confusion
matrix.

    Args:
    - confusion_matrix (torch.Tensor): Confusion matrix of size (2, 2).

    Returns:
    - mcc (float): MCC of the model.
    """
    TP = confusion_matrix[1, 1].item()
    TN = confusion_matrix[0, 0].item()
    FP = confusion_matrix[0, 1].item()
    FN = confusion_matrix[1, 0].item()

    TP = torch.tensor(TP)
    TN = torch.tensor(TN)
    FP = torch.tensor(FP)
    FN = torch.tensor(FN)

    numerator = TP * TN - FP * FN
    denominator = torch.sqrt((TP + FP) * (TP + FN) * (TN + FP) * (TN +
FN)).float()

    mcc = numerator / denominator if denominator != 0 else 0
    return mcc.item()

#####################
# Calibration curve #
#####################

def plot_calibration_curve(model, x_eval, y_eval, subset):

    # Evaluate the model on the test data
    model.eval()
    with torch.no_grad():
        outputs = model(torch.tensor(x_eval.values).float())
        y_pred = outputs.cpu().numpy()
```

```
    fraction_of_positives, mean_predicted_value =
calibration_curve(y_eval, y_pred, n_bins=10)

    # Plot the calibration curve
    plt.plot(mean_predicted_value, fraction_of_positives, marker='o',
label='Neural network', color='gray')

    # Plot a perfectly calibrated line (45-degree diagonal)
    plt.plot([0, 1], [0, 1], linestyle='--', label='Perfectly
Calibrated', color='gray')

    # Add labels and title
    plt.xlabel('Mean Predicted Probability')
    plt.ylabel('Fraction of Positives')
    plt.title('Calibration Curve {}'.format(subset))
    plt.legend()

    # Show the plot
    plt.show()

######################
# Feature importance #
######################

import shap
import torch
import numpy as np

def get_shap_values(model, x_test):
    # Set the model to evaluation mode
    model.eval()

    # Function that SHAP will call to generate model predictions
    def model_predict(data):
        with torch.no_grad():
            tensor_data = torch.tensor(data).float()  # Convert the input
data to tensor
            outputs = model(tensor_data)  # Get model predictions
            return outputs.cpu().numpy()  # Convert predictions to numpy
array

    # SHAP KernelExplainer
    explainer = shap.KernelExplainer(model_predict, x_test)

    # Calculate SHAP values
    shap_values = explainer.shap_values(x_test)

    # If the model has a single output (e.g., regression or binary
classification), `shap_values` should be 2D
```

```
    if isinstance(shap_values, list):
        # If the model has multiple outputs, shap_values will be a list
of arrays
        shap_values = np.array(shap_values[0])  # Taking the first set of
SHAP values

    return shap_values

def plot_feature_importance(x_test, shap_values):
    plt.figure(figsize=(10, 6))
    feature_importance_df = pd.DataFrame({
        'Feature': x_test.columns.to_list(),
        'Importance': np.abs(shap_values).mean(axis=0)
    }).sort_values(by='Importance', ascending=False)

    sns.barplot(x='Importance', y='Feature', data=feature_importance_df,
color='gray')
    plt.title('Global Feature Importance based on SHAP values')
    plt.show()


##############
# CDA curves #
##############

def plot_cda_curves(model, x_test, y_test):
    model.eval()
    with torch.no_grad():
        outputs = model(torch.tensor(x_test.values).float())
        y_pred = outputs.cpu().numpy()

    test_gmv = pd.merge(x_test, y_test, left_index=True,
right_index=True)
    test_gmv["GMV"] = np.round(y_pred)

    clinical_data = pd.read_csv("dataset/Vhir_5005_240124.csv", sep=",")
    clinical_data.rename(columns={"PV":"VP", "SCP":"csPC"}, inplace=True)
    clinical_data.drop(columns=["Unnamed: 0", "SER", "CP"], inplace=True)
    clinical_data["BCN2"] = np.round(clinical_data["BCN2"]/100)

    dca_gmv = dca(
        data=test_gmv,
        outcome='csPC',
        modelnames=['GMV']
    )

    dca_bcn = dca(
        data=clinical_data,
        outcome='csPC',
```

```
        modelnames=['BCN2']
    )

    dca_gmv['model'] = dca_gmv['model'].replace({'all': 'Treat all',
'none': 'Treat none'})
    dca_bcn['model'] = dca_bcn['model'].replace({'all': 'Treat all',
'none': 'Treat none'})

    combined_dca = pd.concat([dca_gmv, dca_bcn])

    # Graficar la curva DCA
    plot_graphs(
        plot_df=combined_dca,
        graph_type='net_benefit',
        y_limits=[0.0, 0.45],
        color_names=["blue", "green", "grey", "orange"]
    )

##############
# CUC Curves #
##############

def plot_CUC_curve(probabilities, statuses, ax):
    """
    Plot the Cumulative Undetected Reclassification (CUC) curve, which
includes
    the Undetected Reclassification (UB) and Saved Biopsy (SB) curves.

    Parameters:
    probabilities (np.array): Array of predicted probabilities.
    statuses (np.array): Binary array (0 or 1) indicating status (1 for
positive, 0 for negative).

    Returns:
    None
    """
    # Ensure probabilities are between 0 and 1, and statuses are binary
    probabilities = np.asarray(probabilities)
    statuses = np.asarray(statuses)

    # Precompute constants
    total_status = np.sum(statuses)
    total_count = len(statuses)

    # Create thresholds
    thresholds = np.arange(0, 100.01, 0.01)

    # Vectorized computation for UB and SB
```

```
    UB_values = np.array([np.sum(statuses[probabilities * 100 < 
threshold]) for threshold in thresholds]) / total_status * 100
    SB_values = np.array([np.sum((probabilities * 100 < threshold) & 
(~np.isnan(statuses))) for threshold in thresholds]) / total_count * 100

    # Plot UB
    ax.plot(thresholds, UB_values, label='Undetected Reclassification', 
color='cornflowerblue', linewidth=1.5)

    # Plot SB
    ax.plot(thresholds, SB_values, label='Saved Biopsy', color='tomato', 
linewidth=1.5)

    # Formatting the plot
    ax.set_xlabel('Threshold Probability (%)')
    ax.set_ylabel('Percentage')
    ax.set_ylim(-5, 100)
    ax.set_xlim(0, 100)
    ax.grid(True, which='both', color='lightgray', linestyle='--', 
linewidth=0.5)
    ax.set_xticks(np.arange(0, 101, 5))
    ax.set_yticks(np.arange(0, 101, 5))

    # Add legend
    ax.legend(loc='upper left', fontsize=10)
```

S3. Model Training

```python
import random
import torch
import optuna
import numpy as np
import torch.optim as optim

from torch import nn
from sklearn.metrics import accuracy_score
from dataset import dataset_training_preparation

############
# Datasets #
############

BSC_data, data, dataloaders, tensors = dataset_training_preparation()

###################
# Seed definition #
###################

def set_seed(seed):
    random.seed(seed)
    np.random.seed(seed)
    torch.manual_seed(seed)
    torch.cuda.manual_seed(seed)
    torch.cuda.manual_seed_all(seed)
    if torch.backends.cudnn.enabled:
        torch.backends.cudnn.benchmark = False
        torch.backends.cudnn.deterministic = True

set_seed(42)

####################
# Model definition #
####################

class SimpleNet(nn.Module):
    def __init__(self, input_size, hidden_size, hidden_size_2, 
output_size):
        super(SimpleNet, self).__init__()

        self.fc1 = nn.Linear(input_size, hidden_size)
        self.fc2 = nn.Linear(hidden_size, hidden_size_2)
        self.fc3 = nn.Linear(hidden_size_2, output_size)

    def forward(self, x):
        x = torch.relu(self.fc1(x))
```

```
        x = torch.relu(self.fc2(x))
        # Apply sigmoid activation for binary classification
        x = torch.sigmoid(self.fc3(x))
        return x

##################
# Weight balance #
##################

def balance_weights(dataset_labels):

    perc_class_1 = torch.sum(dataset_labels) / dataset_labels.numel()
    perc_class_0 = 1 - perc_class_1

    weight_class_0 = 1/(perc_class_0*2)
    weight_class_1 = 1/(perc_class_1*2)

    return [weight_class_0, weight_class_1*1.25]

def batch_weights(batch_labels, weights):
    return batch_labels*weights[1]+(1-batch_labels)*weights[0]

###############################
# Best hyperparameters search #
###############################

def objective(trial):

    # Sample hyperparameters
    input_size = tensors["inputs_tensor_x_train"].shape[1]
    hidden_size = trial.suggest_categorical('hidden_size', [16, 32, 64])
    hidden_size_2 = trial.suggest_categorical('hidden_size_2', [16, 32,
64])
    loss_name = trial.suggest_categorical('loss', ['BCEWithLogitsLoss',
'BCELoss'])
    optimizer_name = trial.suggest_categorical('optimizer', ['Adam',
'SGD', 'Adagrad', 'RMSprop', 'Adadelta', 'AdamW', 'Rprop'])
    output_size = 1  # Binary classification

    model = SimpleNet(input_size, hidden_size, hidden_size_2,
output_size)

    # Calculate class weights
    class_weights = balance_weights(tensors["labels_tensor_y_train"])

    # Choose the optimizer
    lr = trial.suggest_float('lr', 1e-6, 1e-1, log=True)
    if optimizer_name == 'Adam':
        optimizer = optim.Adam(model.parameters(), lr=lr)
```

```
    elif optimizer_name == 'SGD':
        optimizer = optim.SGD(model.parameters(), lr=lr, momentum=0.9)
    elif optimizer_name == 'Adagrad':
        optimizer = optim.Adagrad(model.parameters(), lr=lr)
    elif optimizer_name == 'RMSprop':
        optimizer = optim.RMSprop(model.parameters(), lr=lr, alpha=0.9)
    elif optimizer_name == 'Adadelta':
        optimizer = optim.Adadelta(model.parameters(), lr=lr)
    elif optimizer_name == 'AdamW':
        optimizer = optim.AdamW(model.parameters(), lr=lr)
    elif optimizer_name == 'Rprop':
        optimizer = optim.Rprop(model.parameters(), lr=lr)
    else:
        raise ValueError(f"Unsupported optimizer: {optimizer_name}")

    # Training loop
    num_epochs = 70
    for epoch in range(num_epochs):
        for inputs, labels in dataloaders["dataloader_train"]:
            optimizer.zero_grad()
            outputs = model(inputs)

            if loss_name == 'BCEWithLogitsLoss':
                criterion =
nn.BCEWithLogitsLoss(weight=batch_weights(labels, class_weights))
            elif loss_name == 'BCELoss':
                criterion = nn.BCELoss(weight=batch_weights(labels,
class_weights))
            else:
                raise ValueError(f"Unsupported loss function:
{loss_name}")

            loss = criterion(outputs, labels.float())

            loss.backward()
            optimizer.step()

    # Validation
    model.eval()
    val_loss = 0.0
    val_preds = []
    val_labels = []
    with torch.no_grad():
        for inputs, labels in dataloaders["dataloader_test"]:
            outputs = model(inputs)

            if loss_name == 'BCEWithLogitsLoss':
                criterion =
nn.BCEWithLogitsLoss(weight=batch_weights(labels, class_weights))
```

```
            elif loss_name == 'BCELoss':
                criterion = nn.BCELoss(weight=batch_weights(labels,
class_weights))
            val_loss += criterion(outputs, labels.float()).item()

            preds = torch.sigmoid(outputs).round()
            val_preds.extend(preds.numpy())
            val_labels.extend(labels.numpy())

    # Calculate accuracy
    val_accuracy = accuracy_score(val_labels, val_preds)

    # Combine loss and accuracy into a single metric (weighted sum)
    # Adjust the weights as per your requirements
    combined_metric = val_loss - val_accuracy

    return combined_metric


def get_best_hyperparameters():
    # Setting the seed for Optuna
    sampler = optuna.samplers.TPESampler(seed=42)
    study = optuna.create_study(direction='minimize', sampler=sampler)
    study.optimize(objective, n_trials=100)

    trial = study.best_trial

    print("  Params: ")
    for key, value in trial.params.items():
        print("    {}: {}".format(key, value))

    # Print the best hyperparameters
    best_params = study.best_params
    print('Best Hyperparameters:', best_params)

    # Train the final model using the best hyperparameters
    final_model = SimpleNet(12, best_params['hidden_size'],
best_params['hidden_size_2'], 1)

    if best_params["optimizer"] == 'Adam':
        final_optimizer = optim.Adam(final_model.parameters(),
lr=best_params['lr'])
    elif best_params["optimizer"] == 'SGD':
        final_optimizer = optim.SGD(final_model.parameters(),
lr=best_params['lr'])
    elif best_params["optimizer"] == 'Adagrad':
        final_optimizer = optim.Adagrad(final_model.parameters(),
lr=best_params['lr'])
    elif best_params["optimizer"] == 'RMSprop':
```

```
        final_optimizer = optim.RMSprop(final_model.parameters(), 
lr=best_params['lr'])
    elif best_params["optimizer"] == 'Adadelta':
        final_optimizer = optim.Adadelta(final_model.parameters(), 
lr=best_params['lr'])
    elif best_params["optimizer"] == 'AdamW':
        final_optimizer = optim.AdamW(final_model.parameters(), 
lr=best_params['lr'])
    elif best_params["optimizer"] == 'Rprop':
        final_optimizer = optim.Rprop(final_model.parameters(), 
lr=best_params['lr'])
    else:
        raise ValueError(f"Unsupported optimizer: 
{best_params['optimizer']}")

    return final_model, final_optimizer

###############
# Train model #
###############

def train_model(output_path:str):

    final_model, final_optimizer = get_best_hyperparameters()

    num_epochs = 70
    for epoch in range(num_epochs):
        for inputs, labels in dataloaders["dataloader_train"]:
            final_optimizer.zero_grad()
            outputs = final_model(inputs)
            class_weights = 
balance_weights(tensors["labels_tensor_y_train"])
            final_criterion = nn.BCELoss(weight=batch_weights(labels, 
class_weights))
            loss = final_criterion(outputs, labels)
            loss.backward()
            final_optimizer.step()

    torch.save(final_model, output_path)
```
